# Reduced Radiation Exposure for Face Transplant Surgical Planning Computed Tomography Angiography

**DOI:** 10.1371/journal.pone.0063079

**Published:** 2013-04-26

**Authors:** Kurt Schultz, Elizabeth George, Katherine M. Mullen, Michael L. Steigner, Dimitrios Mitsouras, Ericka M. Bueno, Bohdan Pomahac, Frank J. Rybicki, Kanako K. Kumamaru

**Affiliations:** 1 Toshiba Medical Research Institute USA, Vernon Hills, Illinois, United States of America; 2 Applied Imaging Science Laboratory, Department of Radiology, Brigham and Women's Hospital and Harvard Medical School, Boston, Massachusetts, United States of America; 3 Department of Surgery, Division of Plastic Surgery, Brigham and Women's Hospital, Boston, Massachusetts, United States of America; Royal Melbourne Institute of Technology, Australia

## Abstract

**Objective:**

To test the hypothesis that wide area detector face transplant surgical planning CT angiograms with simulated lower radiation dose and iterative reconstruction (AIDR3D) are comparable in image quality to those with standard tube current and filtered back projection (FBP) reconstruction.

**Materials and Methods:**

The sinograms from 320-detector row CT angiography of four clinical candidates for face transplantation were processed utilizing standard FBP, FBP with simulated 75, 62, and 50% tube current, and AIDR3D with corresponding dose reduction. Signal-to-noise ratio (SNR) and contrast-to-noise ratio (CNR) were measured at muscle, fat, artery, and vein. Image quality for each reconstruction strategy was assessed by two independent readers using a 4-point scale.

**Results:**

Compared to FBP, the median SNR and CNR for AIDR3D images were higher at all sites for all 4 different tube currents. The AIDR3D with simulated 50% tube current achieved comparable SNR and CNR to FBP with standard dose (median muscle SNR: 5.77 vs. 6.23; fat SNR: 6.40 vs. 5.75; artery SNR: 43.8 vs. 45.0; vein SNR: 54.9 vs. 55.7; artery CNR: 38.1 vs. 38.6; vein CNR: 49.0 vs. 48.7; all p-values >0.19). The interobserver agreement in the image quality score was good (weighted κ = 0.7). The overall score and the scores for smaller arteries were significantly lower when FBP with 50% dose reduction was used. The AIDR3D reconstruction images with 4 different simulated doses achieved a mean score ranging from 3.68 to 3.82 that were comparable to the scores from images reconstructed using FBP with original dose (3.68–3.77).

**Conclusions:**

Simulated radiation dose reduction applied to clinical CT angiography for face transplant planning suggests that AIDR3D allows for a 50% reduction in radiation dose, as compared to FBP, while preserving image quality.

## Introduction

Facial allograft transplantation restores form and function in patients with severe deformities [1] and is rapidly gaining acceptance for complex craniofacial reconstruction. Vascular anastomosis is critical to technical success, and thus pre-operative vascular mapping [2] plays a large role for a safer procedure [3]. Both Computed Tomography (CT) and magnetic resonance methods [4], [5] have been studied for surgical planning [6]. Because CT angiography (CTA) enables high image quality 3- or 4-dimensional vascular assessments, it is preferred over catheterization or other noninvasive methods to delineate the presence, course, caliber, and contrast enhancement of the recipient's arteries and veins with relationships to other craniofacial landmarks. To our knowledge, there is a single report of radiation exposure for face transplant CTA [2]. However, given the rapid growth of face transplant programs and multiple CT studies that patients will undergo as screening and follow-up, consideration of radiation doses for comprehensive CT examination is prudent.

Iterative reconstruction methods are algorithms that reduce image noise by iteratively comparing the acquired noise to a modeled projection [7], [8], and have been applied to many CT applications, including CTA [9]–[14]. Reduced image noise achieved by iterative reconstruction enables lower tube currents, resulting in reduced radiation dose [7], [10], [15]–[19]. In general, each algorithm is specific to a CT vendor as the software is applied to sinogram data. One of the most recent algorithms is an Adaptive Iterative Dose Reduction (AIDR) algorithm in Three-Dimensions (AIDR3D) [20] that works in both the raw and image domains.

To date, imaging reports for face transplantation have focused on wide area detector CT, and there are no known data evaluating iterative reconstruction as a possible option for radiation dose reduction. Because clinical trial mandates strict adherence to protocol for these patients, retrospective evaluation is favored before implementing a practice change. This can be achieved by simulating the reduced tube current using a mathematical addition of image noise to the CT sinogram data. The purpose of this study was to test the hypothesis that wide area detector face transplant surgical planning CTA images with simulated lower radiation dose and iterative reconstruction are comparable in image quality to images with standard tube current from our institution that are reconstructed using filtered back projection.

## Materials and Methods

### Subjects

We retrospectively evaluated 4 patients who signed written informed consent approved by our Institutional Human Research Committee. These patients voluntarily enrolled in clinical trial NCT01281267, and are documented in the US Army Medical Research and Materiel Command's Human Research Protection Office. Brief clinical history of the 4 patients is as follows.

#### Case 1

30-year-old man who was involved in a motor vehicle accident, resulting in a high voltage electrical injury to his face. After multiple conventional reconstructive surgeries, he underwent full face transplantation.

#### Case 2

25-year-old man who had catastrophic loss of facial tissues after high voltage injury. After 20 procedures including multiple flaps covered with skin grafts, other surgical options were exhausted and the patient underwent full face transplantation.

#### Case 3

35-year-old man who had a gunshot wound that shattered his mandible and maxilla. He underwent multiple reconstructions resulting in substantial facial deformity and was considered for face transplantation.

#### Case 4

28-year-old man who had a gunshot wound to his mid face. After multiple conventional reconstructions, he was considered for face transplantation.

### CT acquisition

All patients were imaged with a single-volume 320×0.5 mm detector row CT (Aquilion ONE, Toshiba Medical Systems Corporation, Tochigi-ken, Japan). The gantry rotation time was 500 milliseconds; images were reconstructed at 0.5 mm increments. After a 20 mL test bolus to plan the contrast enhancement timing, the dynamic study was performed using a 60 mL intravenous iodinated contrast medium (iopamidol 370 mg iodine per milliliter, Isovue-370, Bracco Diagnostics, Princeton, New Jersey) administered via a power injection (Empower CTA, Acist Medical, New York) system at contrast flow rates of 4 to 6 mL/s, followed by 40 mL normal saline. For the dynamic study, the tube voltage of 80 kV and the mAs of 155 were used for all patients. Intermittent dynamic volumes (0.50-second gantry rotation) included 18–24 volumes, for phases from arterial uptake through venous return. Scanner output data (the extended dose length product) were used to estimate the radiation dose. For conversion to estimated effective dose (millisievert), the field of view (FOV) exposing the neck used k = 0.0059 mSv/mGy-cm and the FOV exposing the head used k = 0.0023 mSv/mGy-cm [2].

### Image data reconstruction

For all 4 patients, an experienced radiologist identified one arterial phase and one venous phase with the ideal contrast enhancement for surgical planning. Then, a database of 96 reconstructions (24 for each of the 4 patients) was created from the raw data. Sinogram data was retrieved from the scanner systems and archived using a raw data server (Toshiba Medical Systems Corporation, Japan) equipped to add noise to the sinograms with a noise addition tool. Both the raw data server and the noise addition software were used under a research agreement with the manufacturer. This noise simulation methodology allows for accurate determination of the noise based on direct measurements from the detector, data acquisition system performance, and the x-ray generation. The noise tool injects a combination of Poisson noise for photon statistics and Gaussian electronic noise into the raw projections based on the desired reduction in tube current to be simulated. The noise added data is then used to create the projections after corrections and logarithmic conversion.

The 24 reconstructions were divided into 8 each for 3 sets of CT sinograms: non-contrast, best arterial phase, and best venous phase acquisitions. Four of the 8 reconstructions used the original exposure settings and the recommended manufacturer filtered back projection (FC41) kernel for soft tissue display of facial anatomy. In 3 of these 4 filtered back projection reconstructions, CT noise was added to the raw data to simulate image quality that would have been obtained with the mAs of 75%, 62%, and 50% of that used clinically (155 mAs). The remaining 4 of 8 reconstructions, after applying the same simulated mAs reductions, used AIDR3D. AIDR3D works in both the raw and image domains and is fully integrated into the 320×0.5 mm detector row CT acquisition workflow.

### Objective image quality assessment

To compare attenuation and image noise among the reconstructed data sets, region-of-interest (ROI) measurements of mean and standard deviation Hounsfield Units (HU) were obtained in the masseter muscle, anterior fat tissue to the masseter region, air in the sphenoidal sinus, carotid artery for the best arterial phase, and the internal jugular vein for the best venous phase; this ROI measurement was repeated 5 times. Signal-to-noise ratio (SNR) was calculated at muscle (non-contrast), fat (non-contrast), artery (arterial phase), and vein (venous phase) by dividing the absolute mean value within the ROI by the standard deviation in air. To compare contrast-to-noise ratio (CNR) among the different reconstructions, the difference in mean HU between the vessel (i.e., carotid artery or internal jugular vein) and muscle were divided by the standard deviation in air.

### Subjective image quality assessment

To evaluate potential differences in diagnostic image quality of vessels among the reconstructed data sets, the following vessels considered important for face transplantation were assessed: internal and external carotid artery, lingual artery, facial artery, superior thyroid artery, superficial temporal artery, internal and external jugular vein, and common facial vein. Before interpretation by two cardiovascular imagers with 1 and 2 years of experience, respectively, in the interpretation of face transplant surgical planning, who were blinded to the image reconstruction technique, one radiologist reviewed all images and medical records to determine the vascular anatomy of each patient. This included surgical findings for those patients who underwent surgery. Vessels that were absent, either from injury or prior reconstructions, were excluded from analyses. For each patient, the image interpretation was performed on one side that was randomly selected for each patient. The two readers independently ranked overall image quality at each vessel, using a 4-point scale based on vessel sharpness, image noise, streak or other artifacts where 4 =  excellent, no artifact; 3 =  good, mild artifact; 2 =  acceptable, moderate artifact present but images still interpretable; and 1 =  unevaluable with severe artifacts rendering interpretation impossible.

### Statistical analysis

The SNR and CNR among different reconstructions were summarized using boxplots. For the subjective image quality scores, interobserver agreement was evaluated with a weighted Cohen's kappa test (weighting of 0.8 for the closest score) with the following scale: less than 0.20, poor; 0.21–0.40, fair; 0.41–0.60, moderate; 0.61–0.80, good; and 0.81–1.00, excellent agreement. The Friedman test with a post-hoc multiple comparisons evaluated the statistical difference in image quality score (the average of two readers) and in the SNR and CNR among the different reconstruction methods. Statistical analyses were performed using STATA version 10.1 (Stata Corp., College Station, TX).

## Results

All CTA studies were acquired without complication. The total imaging time for each study was under 45 minutes. The estimated radiation exposure was 7.08, 7.03, 6.54, and 9.47 mSv for patients 1–4, respectively.

### Objective image quality assessment

In 478 of the total 480 (99.6%) individual measurements (i.e., 4 SNR (muscle, fat, artery, and vein) and 2 CNR (artery and vein) measurements for 4 patients with 4 different mAs, all repeated 5 times), AIDR3D achieved a higher value than that from the FBP image with corresponding mAs. Compared to FBP images acquired with original dose, AIDR3D images with 75%, 62%, and 50% mAs achieved a superior value in 94% (113/120), 62.5% (75/120), and 70% (84/120) of the individual measurements.

**Figure 1:**
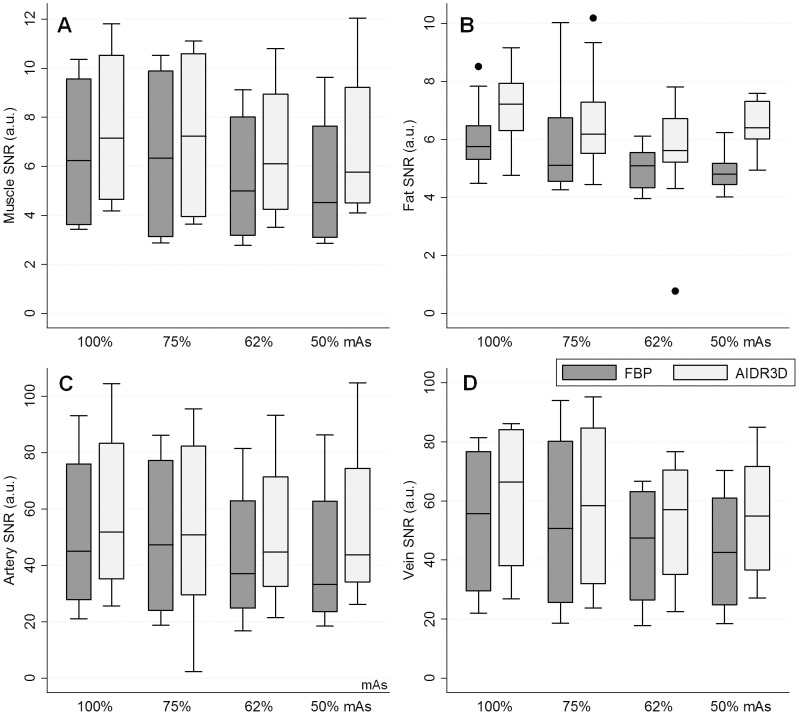
Boxplots of signal-to-noise ratio (SNR) for each reconstruction. A- Muscle. B- Fat. C- Artery. D- Vein.

**Figure 2:**
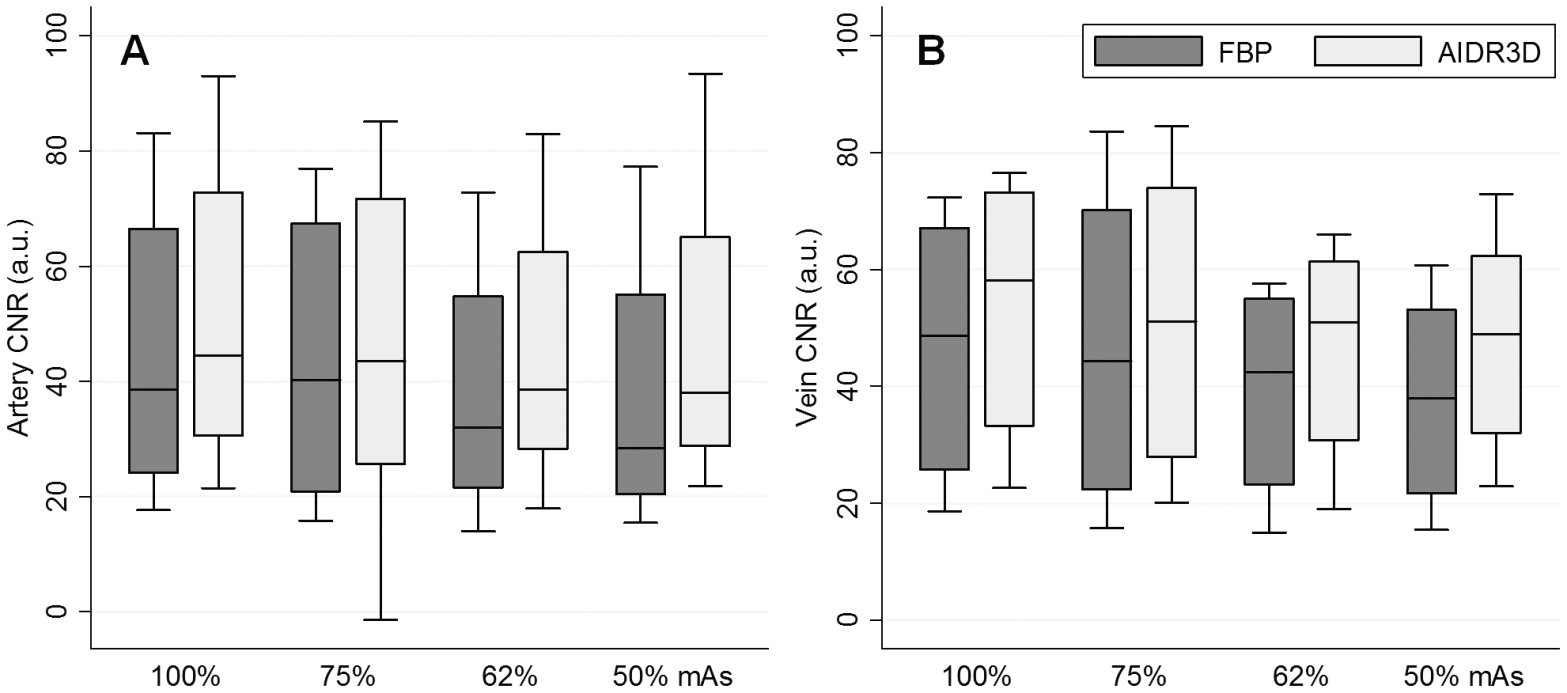
Boxplots of contrast-to-noise ratio (CNR) of each reconstruction. A- Artery. B- Vein.

When considering the median SNR (Figure 1) and CNR (Figure 2), AIDR3D achieved significantly higher values than FBP at all 100%, 75%, 62%, and 50% mAs settings, with statistically significant differences (all p-values ≤0.0015). Images with the simulated 50% reduction in mAs and with AIDR3D reconstruction had similar SNR and CNR as those with 100% mAs and FBP reconstruction (FBP with 100% mAs vs. AIDR3D with 50% mAs, median muscle SNR: 6.23 vs. 5.77; fat SNR: 5.75 vs. 6.40; artery SNR: 45.0 vs. 43.8; vein SNR: 55.7 vs. 54.9; artery CNR: 38.6 vs. 38.1; vein CNR: 48.7 vs. 49.0, all p-values >0.19).

### Subjective image quality assessment

Three anatomically absent vessels were excluded from evaluation: the facial artery for patient 2 and 3 and the external jugular vein for patient 3. For the remaining 264 vessels (22 vessels for 8 different reconstructions), the interobserver agreement between the two readers was good (weighted kappa value  = 0.7), with 84% (222/264) of vessels being identical between readers.

Considering the mean image quality score for the two readers (Table 1), the images reconstructed with FBP and simulated 50% reduction of mAs showed the lowest quality score among the methods at all vessels, with a significantly (p<0.001) lower overall score for smaller arteries (lingual, superior thyroid, and superficial temporal arteries), all arteries, and all veins (3.36 vs. 3.50–3.71 for smaller arteries, 3.45 vs. 3.61–3.80 for all arteries, and 3.59 vs. 3.64–3.77 for all veins, respectively). Images reconstructed with AIDR3D at all four mAs had comparable image quality as the images with original mAs and FBP reconstruction, even for the smaller arteries (AIDR3D with 4 different mAs vs. FBP with original mAs  = 3.68–3.71 vs. 3.68 for smaller arteries). Images from AIDR3D with reduced mAs achieved higher mean scores for the superior thyroid and superficial temporal artery when compared to FBP images reconstructed with the original mAs (Table 1).

**Table 1:** Mean image quality score (average of two readers) for each of the eight image reconstruction strategies at each vessel.

			FBP				AIDR3D				
			mAs				mAs				p-value
		No.	100%	75%	62%	50%	100%	75%	62%	50%	
Large arteries	Internal carotid	4	4.00	3.75	4.00	3.63	3.88	3.88	4.00	4.00	-
	External carotid	4	4.00	3.75	3.88	3.63	3.88	4.00	4.00	4.00	-
	Overall	8	4.00	3.75	3.94	3.63	3.88	3.94	4.00	4.00	0.798
Small arteries	Facial	2	4.00	3.75	3.75	3.75	4.00	4.00	4.00	4.00	-
	Lingual	4	3.88	3.63	3.75	3.50	3.75	3.75	3.88	3.75	-
	Superior thyroid	4	3.50	3.25	3.38	3.13	3.50	3.50	3.63	3.50	-
	Superficial temporal	4	3.50	3.50	3.25	3.25	3.75	3.63	3.50	3.63	-
	Overall	14	3.68	3.50	3.50	3.36*	3.71	3.68	3.71	3.68	<0.001
	Artery overall	22	3.77	3.61	3.64	3.45*	3.80	3.77	3.82	3.80	<0.001
Veins	Internal jugular	4	4.00	3.88	3.88	3.88	3.88	3.88	4.00	4.00	-
	External jugular	3	3.67	3.67	3.67	3.50	3.67	3.67	3.67	3.67	-
	Common facial	4	3.63	3.38	3.50	3.38	3.50	3.50	3.63	3.63	-
	Vein overall	11	3.77	3.64	3.68	3.59*	3.73	3.68	3.77	3.77	<0.001

* Significantly lower compared to at least one other reconstruction. “-” indicates no statistical comparison due to a small number in each subgroup.

**Figure 3:**
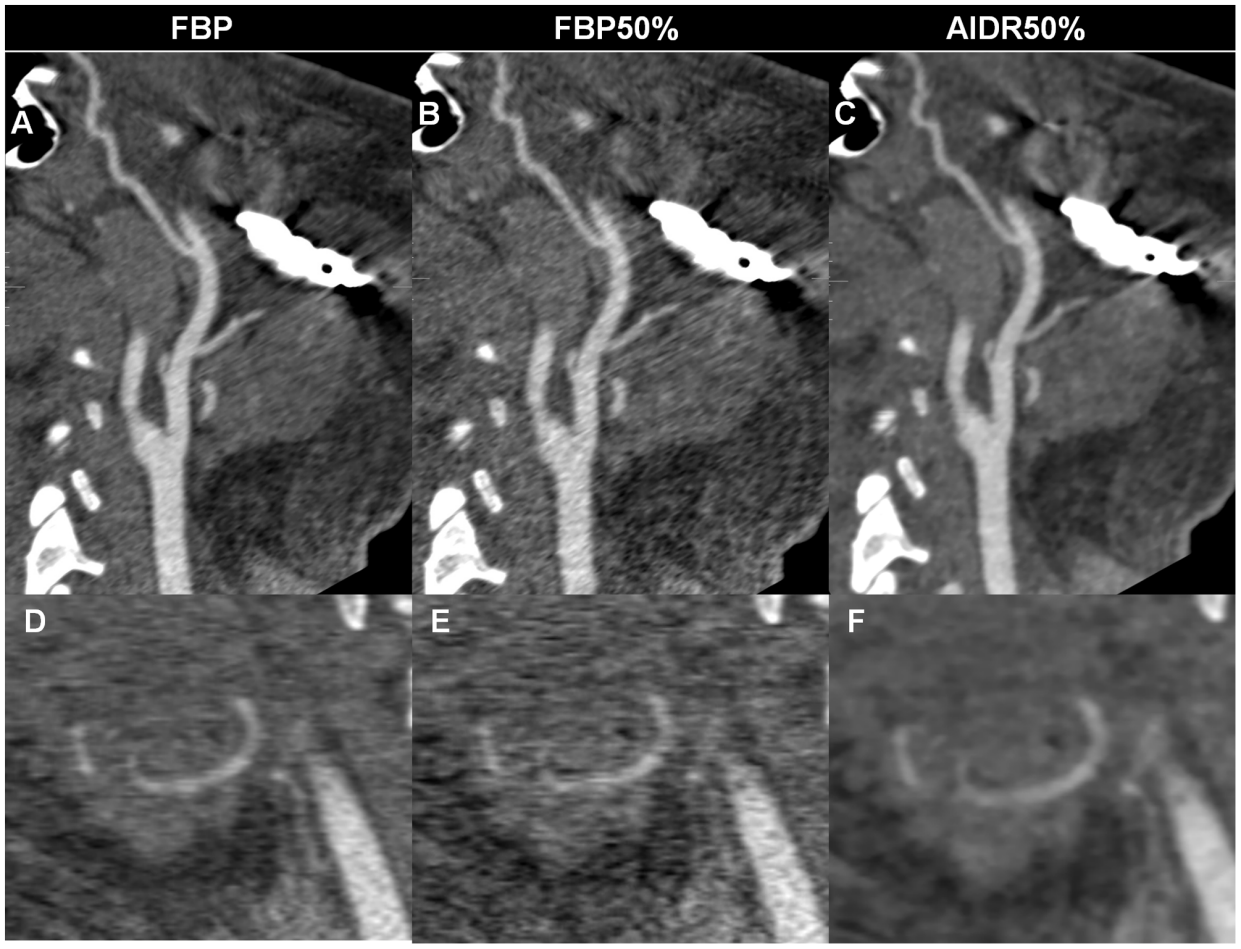
Representative images with FBP and AIDR3D reconstructions. Image noise increases on images reconstructed using FBP with original tube current (A, D) and simulated 50% dose reduction (B, E), especially around the metal in the mandible (A, B), while images reconstructed using AIDR3D with simulated 50% dose reduction (C, F) achieve reduced artifacts and noise. The right lingual artery (D–F) is poorly delineated in the image with FBP and simulated 50% dose reduction (E).

**Figure 4:**
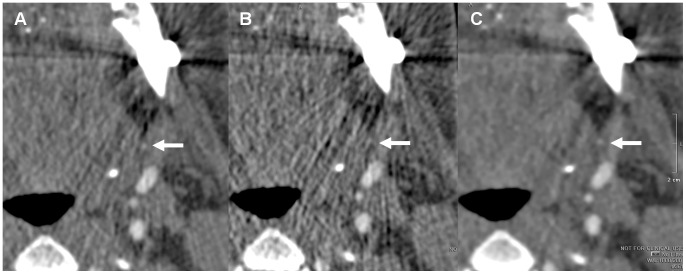
Representative images with FBP and AIDR3D reconstructions. The branch of the left facial artery (arrow) is clearly depicted on the image reconstructed using AIDR3D with simulated 50% dose reduction (C), while it is obscured on the image reconstructed using FBP with original dose (A) and is hard to detect on the image using FBP with simulated 50% dose reduction (B) due to streak artifacts.

Figure 3 and 4 show the representative images for different reconstruction methods from the same patient.

## Discussion

Face transplant candidates are in general relatively young, are of good health, and will require multiple CT scans over their lifetime. In addition, less aggressive immunosuppression has decreased the long-term risks [21]. Combined with excellent outcomes [1], the long life-expectancy of this growing population challenges the imaging protocols to optimize radiation dose while still achieving excellent image quality for surgical planning. Since January 2012, in the United States alone, several new face transplant programs have been developed, and there is a growing need to standardize low radiation dose imaging.

The CTA literature for surgical planning of face transplant to date uses multiphase wide area detector technology [2] to determine those vessels best suited for surgical anastomoses [6]. However, the multiple acquisitions over time have greater exposure than a fewer number of static acquisitions. There are two at risk organs from the increase of radiation dose, the thyroid gland and the orbits. The cumulative dose to the thyroid should be monitored to avoid increasing risk of thyroid cancer [22], [23]. It is also important to limit the radiation to the globes for patients with at least partial vision to avoid cataract formation [24].

AIDR3D was introduced to reduce patient radiation exposure while maintaining image quality, and it has been used in the chest and abdomen [25], [26], the coronary arteries [27]–[29], and for the liver perfusion imaging [30]. The adaptive photon reduction is applied directly to the photon count values. In our experience this reduces streak artifacts, an important part of surgical planning because face transplant candidates have substantial metal from their injury, prior interventions, or typically both [6], [31]. The algorithm has been designed to work in both the three dimensional (3D) raw data and reconstruction domains. Within the raw data domain, adaptive photon noise reduction is achieved by using a statistical noise model and a scanner model. The statistical modeling characterizes both electronic and quantum noise patterns in projection space. The scanner model analyzes the physical properties of the CT system at the time of acquisition, using a 3D smoothing filter that accounts for photons of adjacent rows as well as detector channels and views. In the image space, an iterative technique optimizes a balance between noise suppression and preservation of fine details. A weighted blending with FBP is used; this maintains granularity. Figures 3 and 4 illustrate the reduction in streak artifacts near important vessels needed for face transplantation surgical planning.

Human subject guidelines for face transplantation at our institution do not allow for multiple surgical planning CT acquisitions in the same patient. Thus, to evaluate the new iterative reconstruction technology, a validated [32] CT noise addition software tool developed by the manufacturer was used to directly compare quality among images that depict the same anatomy through simulation of a lower tube current [33]. Noise addition tools have been effectively used to evaluate the effects of dose reduction, primarily outside the head and neck [34]–[36].

Simulated radiation dose reduction for face transplant planning CTA revealed suboptimal image quality for FBP reconstruction images when the tube current was reduced by 50%, especially for smaller vessels such as the lingual, superior thyroid, or superficial temporal arteries. Delineation of these vessels is essential because they could be the target of anastomosis [37]. Images reconstructed with AIDR3D demonstrated maintained image quality for these smaller vessels when the simulated tube current was reduced by 50%. Based on the current data, we have recently changed face transplant surgical planning CT protocol at our institution to include AIDR3D. Future studies are planned to confirm excellent image quality for our patients with an estimated effective radiation doses of less than 5 mSv.

Our study limitations include a small patient cohort. However, future imaging will include AIDR3D on a prospective basis, and we will then be able to expand the patient cohort, and correlate the image findings with those at surgery.

## Conclusions

Using simulated radiation dose reduction for face transplant planning CTA, AIDR3D maintained image quality with a 50% reduction in radiation dose.
